# *Scenopinusjerei*, a new species of window fly (Diptera, Scenopinidae) from Finland

**DOI:** 10.3897/zookeys.1059.70085

**Published:** 2021-09-10

**Authors:** Jaakko Pohjoismäki, Antti Haarto

**Affiliations:** 1 University of Eastern Finland, Department of Biology, P.O. Box 111, FI-80101 Joensuu, Finland University of Eastern Finland Joensuu Finland; 2 Zoological Museum, Biodiversity Unit, University of Turku, FI-20014 Turku, Finland University of Turku Turku Finland

**Keywords:** DNA barcoding, synonyms, taxonomy

## Abstract

A new species of window fly (Diptera: Scenopinidae), *Scenopinusjerei***sp. nov.**, with characteristic bicoloured legs and completely black halteres, is described from Finland. To exclude potential previously named species, a survey of the relevant type specimens as well as original descriptions of the Palearctic and Nearctic *Scenopinus* species has been conducted, including old *Scenopinusfenestralis* (Linnaeus) synonyms. *Scenopinusjerei***sp. nov.** is likely to be an overlooked, boreal forest specialist living in the nests of cavity-nesting birds. An identification key to the European species is provided.

## Introduction

Window flies (Diptera: Scenopinidae) are a small family of primitive flies belonging to the therevoid clade of the Asiloidea superfamily (Winterton & Ware, 2015). The family has a cosmopolitan distribution, with more than 420 described species in 25 genera. Scenopinidae consists of three subfamilies, Caenotinae (1 genus), Proratinae (6 genera), and Scenopininae (18 genera) (Yeates 1992; Winterton and Gaimari 2017), with only the last one being present in Europe. For a more detailed description of the family and subfamilies, including their biology and classification see Winterton and Gaimari (2017). In total, 18 species have been known to occur in Europe (Kelsey 1969, 1981; Narchuk 1988; Carles-Tolrá 1999; Bystrowski et al. 2021): *Caenoneuranigra* Kelsey, 1969, *Scenopinusalbicinctus* (Rossi, 1794), *S.bouvieri* (Seguy, 1921) (not listed in Fauna Europaea – see Kelsey 1969; Narchuk 1988), *S.bulbapennis* Kelsey, 1969, *S.canarius* Kelsey, 1969, *S.efflatouni* Kelsey, 1969, *S.fenestralis* (Linnaeus, 1758), *S.glabrifrons* Meigen, 1824, *S.gobiensis* Kelsey, 1981, *S.griseus* (Kröber, 1913), *S.halteralis* Frey, 1936, *S.lesinensis* Strobl, 1902, *S.niger* (De Geer, 1776), *S.oldenbergi* (Kröber) (not listed in Fauna Europaea – see Kelsey 1969; Narchuk 1988), *S.retuertensis* Carles-Tolra, 2001 *S.unifasciatus* (Krober, 1913), *S.verrucosus* Carles-Tolra, 2001, and *S.vitripennis* Meigen, 1824. In addition, *S.phaidimos* Kelsey, 1969 is present in Turkey and might be expected to occur in the eastern Mediterranean.

The greatest diversity of Scenopinidae is in the arid regions of the world (Winterton and Ware 2015), which is also reflected by the fact that the majority of the European species are present in the Mediterranean countries and Macaronesian islands. Only *Scenopinusfenestralis*, *S.glabrifrons*, *S.niger*, and *S.vitripennis* extend their range to central and northern Europe. Three species are known from Finland (Kahanpää et al. 2014), *Scenopinusfenestralis*, *S.niger*, and a relatively common, but previously unnamed species close to *S.fenestralis*. The unnamed species was originally reported as *S.vitripennis* (Haarto 2000), but a closer examination proved this to be a misidentification (Kahanpää and Winqvist 2005; Kahanpää et al. 2014).

In this paper we finally provide a formal description of the previously undescribed species as well as information about its distribution and biology. We focus on differentiating the species from *Scenopinusfenestralis* and *S.vitripennis*, as these are most likely to be confused with the new species due to the variability of all three species as well as because of the diagnostic characters used in older literature. We also compared it with written descriptions of other known species in the Holarctic and the old synonyms of *Scenopinusfenestralis* to rule out existing names for the candidate species. Finally, a key for the identification of the European *Scenopinus* species is provided.

## Materials and methods

### Material examined

Apart for the two old museum specimens of *Scenopinusvitripennis*, most of the examined material were collected relatively recently by the authors and their close associates. Except for the reared specimens, majority of the examples have been collected indoors (see notes) directly to vials and killed by freezing, ethyl acetate or potassium cyanide prior to mounting them on entomological pins.

Label data of newly collected specimens are given verbatim using the following symbols: / end of a line and beginning of the next; // end of label and beginning of the next (from top to bottom on the same pin). The specimens are deposited in the following collections and are indicated with the given acronym in the text:

**AHC** Private collection of Antti Haarto, Mynämäki, Finland

**JPC** Private collection of Jaakko Pohjoismäki, Joensuu, Finland

**MIZ** Museum and Institute of Zoology, Polish Academy of Sciences, Warszawa, Poland

**MZH** Finnish Museum of Natural History, Zoological Museum, University of Helsinki, Helsinki, Finland

**MZT** Zoological Museum of the University of Turku, Turku, Finland

**SMNS** Staatliches Museum für Naturkunde Stuttgart, Germany

Male terminalia were dissected and prepared for examination essentially as described by O’Hara (2002). The dissected terminalia are preserved in glycerol in a small plastic vial pinned together with the specimen. Location abbreviations refer to the geographical provinces (e.g., Kb = Karelia borealis, explained when mentioned for the first time, see https://laji.fi/theme/emk for more details) and coordinates (NNNN:EEEE) on the labels are mostly given in the old national Finnish map grid coordinate system (YKJ; see Ollikainen and Ollikainen 2004), which is still in common use for biological sampling. As the first number of the longitude coordinate is always 3, this is often left out in the collection labels to save space. Sampling coordinates in decimal degrees as well as additional notes or commentaries about the specimen are given in brackets.

#### *Scenopinusfenestralis* (Linnaeus, 1758)

**Finland**. (6♂♂, 8♀♀) 1♂(dissected): Fennia Kb [Karelia borealis – north Karelia]: Liperi/ Kontkala 6950:3616 [62.6257, 29.3264]/ 19.7.2014/ Ali Karhu leg. [JPC]; 1♀: Same collection data [JPC, DNA barcoded JP01083]; 1♀: Finland, Kb: Ilomantsi/ Kelovaara 70008:36825 [63.0638, 30.6144]/ 25.6.2016/ J. Pohjoismäki leg. [JPC]; 1♀: Finland, Sa [Savonia australis – south Savo]: Taipalsaari/ Riihilahti 6778:3564 [61.1055, 28.1867]/ 21.7.2015/ J. Pohjoismäki leg. [JPC, DNA barcoded JP01084]; 1♂: Finland, Ta [Tavastia australis – south Häme]: Orivesi/ Siitama 6835:3354 [61.5946, 24.2496]/ 11.7.2009/ J. Pohjoismäki leg. [JPC]; 1♀: Finland, Ok [Ostrobottnia kajanensis – Kainuu region]: Sotkamo/ Laukkala, 7114:3565 [64.1192, 28.3340]/ 1.7.2005/ J. Pohjoismäki leg. [JPC]; 1♀: Finland, Ab [regio Aboensis – Turku region]: Mynämäki/ Perkko 6733:3222 [60.6105, 21.9209]/ 22.7.2011/ A. Haarto leg. [MZH, DNA barcoded, MZH_HP.392]. 1♂: Finland, Ab: Mynämäki/ Perkko 6733:3222 [60.6105, 21.9209]/ 13.6.2009/ A. Haarto leg.// SCENOPINIDAE/ Scenopinus/ fenestralis (L.)/ det. A. Haarto 2009/ AHa09–000593 [MZT]; 1♀: Finland, Ab: Mietoinen/ Perkko 6733:[3]222 [60.6105, 21.9209]/ 17.7.2003/ A. Haarto leg.// SCENOPINIDAE/ Scenopinus/ fenestralis (L.)/ det. A. Haarto 2008/ AHa08–001324 [AHC]; 1♀: Finland, Ab: Mietoinen/ Perkko 6733:[3]222 [60.6105, 21.9209]/ 17.7.2003/ A. Haarto leg.// SCENOPINIDAE/ Scenopinus/ fenestralis (L.)/ det. A. Haarto 2008/ AHa08–001324 [AHC]; 1♂: Finland, Ab: Mietoinen/ Perkko 6733: [3]222 [60.6105, 21.9209]/ / 16.5.2004/ A. Haarto leg.// SCENOPINIDAE/ Scenopinus/ fenestralis (L.)/ det. A. Haarto 2021/ AHa21–000589 [AHC]; 1♂: Finland, Ab: Mietoinen/ Perkko 6733: [3]222 [60.6105, 21.9209]/ 5.6.2004/ A. Haarto leg.// SCENOPINIDAE/ Scenopinus/ fenestralis (L.)/ det. A. Haarto 2021/ AHa21–000590 [MZT]; 1♂: Finland, EP [Etelä-Pohjanmaa]: Isokyrö/ Orisberg 6983: [3]265 [62.8744, 22.3795]/ 7.7.1999/ A. Haarto leg.// SCENOPINIDAE/ Scenopinus/ fenestralis (L.)/ det. A. Haarto 1999 [AHC]; 1♀: Same collection and determination data [AHC];

#### *Scenopinusglabrifrons* Meigen, 1824

**Germany**: 2♀♀: Germany/ Hessen, Friedberg/ Ockstadt 50.3319, 8.7208 [Geographic coordinate]/ 13.6.2010/ J. Pohjoismäki leg. [JPC]

**Greece**: 2♀♀: GR CRETE Chania/ Thymia 35.4106, 24.0440 [Geographic coordinate]/ 5.-6.vi.2019/ J. Pohjoismäki leg. [JPC]

#### *Scenopinusjerei* sp. nov.

**Finland**: 6♂♂, 4♀♀. See the type material below for details.

#### *Scenopinusniger* (De Geer, 1776)

**Finland**: (4♂♂, 7♀♀) 2♀♀: Finland, Sa: Kouvola, 674–679:347–350 [60.7686–61.2184, 26.4495–27.0000]/ e.l. 2018 ex *Strixaluco* nest box. / M. Mutanen leg. [JPC]; 1♂: Finland, Sa: Taipalsaari/ Riihilahti 6778:3564 [61.1055, 28.1867]/ 21.7.2015/ J. Pohjoismäki leg. [JPC, DNA barcoded, JP01085]; 1♀: Finland, Ta: Tampere/ Rantaperkiö 6822:3327 [61.4669, 23.7541] / 26.6.2009/ J. Pohjoismäki leg. [JPC]; 1♀: Finland, Ab: Mynämäki/ Perkko 6733:3222 [60.6105, 21.9209]/ 12.6.2011/ A. Haarto leg. [MZH, DNA barcoded, MZH_HP.185]; 1♀: Finland, V [Varsinais-Suomi]: Turku Hirvensalo/ Rauhala 6707:[3]233/ 22.5.1996/ A. Haarto leg.// SCENOPINIDAE/ Scenopinus/ niger (DeGeer)/ det. A. Haarto [AHC];] ; 1♂: Finland, V: Turku Hirvensalo/ Rauhala 6707: [3]233 [60.3854, 22.1559]/ 7.6.1996/ A. Haarto leg.// SCENOPINIDAE/ Scenopinus/ niger (DeGeer)/ det. A. Haarto [AHC]; 1♂1♀: Finland, V: Turku Hirvensalo/ Rauhala 6707: [3]233 [60.3854, 22.1559]/ 7.6.1996/ A. Haarto leg.// SCENOPINIDAE/ Scenopinus/ niger (DeGeer)/ det. A. Haarto [MZT]; 1♂: Finland, Ab: Mynämäki/ Perkko 67333:32223 [60.6105, 21.9209]/ 22.5.2017/ A. Haarto leg.// SCENOPINIDAE/ Scenopinus/ niger (De Geer)/ det. A. Haarto 2017/ AHa17–001063 [AHC]; 1♀: Finland, V: Kaarina/ Kuusisto Rövarholm/ 9.7.1998/ A. Haarto leg.// SCENOPINIDAE/ Scenopinus/ niger (De Geer)/ det. A. Haarto 2008/ AHa08–001326 [AHC].

#### *Scenopinusvitripennis* Meigen, 1824

**Germany**: 1♂: Scen. glabrifrons/ Württbg Meig. ?/ v.Roser 1872 [handwritten]// Scenopinus/ vitripennis Meig./ det. L.P. Kelsey 1964 [SMNS]. Examined from high resolution photographs. See the discussion regarding the identity of this specimen.

**Poland**: 1♀: Warszawa [barely visible]/ 14.vii.1953 r./ leg. R. Trojan// Omphrale ♀/ vitripennis (Meig)/ P. Trojan det. 1954. [MIZ]. Examined from high resolution photographs.

### Classification and terminology

The classification follows Herting and Dely-Draskovits (1993). The morphological terminology used in this study follows Cumming and Wood (2017), except for the features of male terminalia, where Winterton and Gaimari (2017) is used.

### Microscopy and imaging

The images were taken with a Leica Z6APO stereomicroscope and a Leica DFC450c (5MPix) camera, MSV266 motorised focus and using the Leica Application Suite 4.6.0 software for Z-axis stacking. Images were cropped, colour- and contrast-enhanced but not manipulated otherwise.

### DNA extraction, PCR, and sequencing

Cytochrome oxidase subunit 1 (*COI*) DNA barcoding was performed as a part of the Tachinidae project of Finnish Barcode of Life initiative (FinBoL). The 5´-terminal part of *COI* was amplified using the routine barcoding primers LepF1 and LepR1 (Hebert et al. 2004). The sample identifiers in the barcode of life database (BOLD) are given for each barcoded specimen.

### Sequence comparisons and COI tree

Sequence comparisons were performed using MUSCLE alignment (Edgar 2004) and Bayesian inference phylogenetic tree generated using MrBayes 3.2. (Ronquist et al. 2012), applying GTR substitution model with gamma-distributed rate variation across sites and a proportion of invariable sites, and 1,000,000 MCMC generations. The tree was visualised using FigTree 1.4.4. (Rambaut 2009).

## Results

We report here a new species of window flies, *Scenopinusjerei* sp. nov. from Finland based on the following material and diagnostic characters.

### Scenopinus jerei sp. nov.

Taxon classificationAnimaliaDipteraScenopinidae

E926E62F-434C-541C-8825-3CA249C0E706

http://zoobank.org/7BBF06EE-AA55-42A8-B2DF-88FC4640AFA0

**Figure 1.**
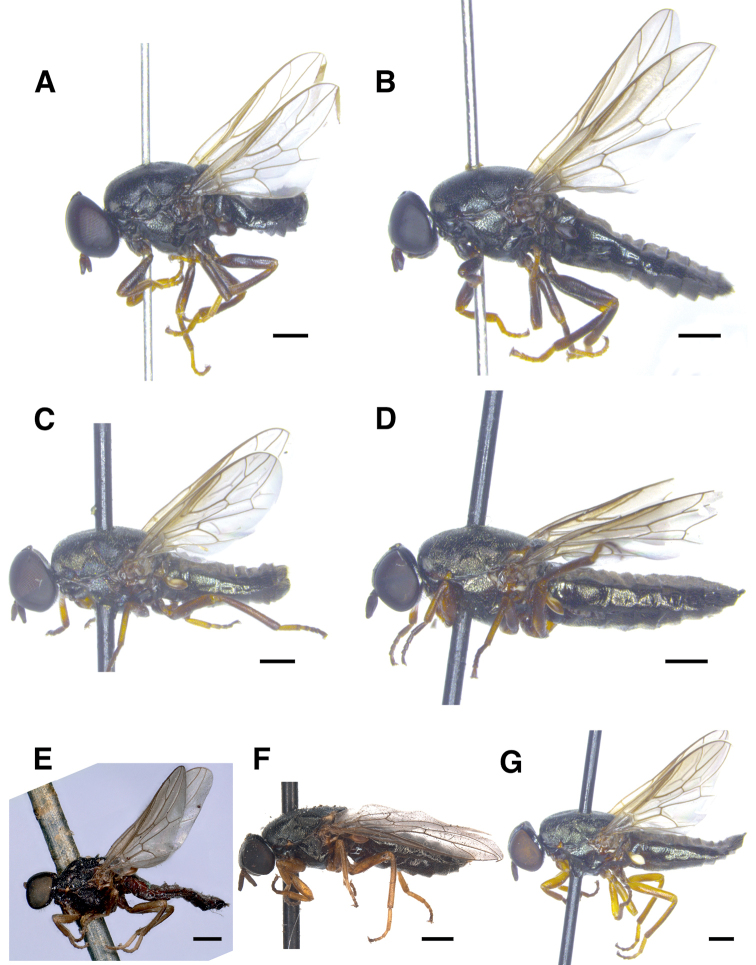
Habitus of northern European *Scenopinusfenestralis* group species of Scenopinidae**A** holotype male of *Scenopinusjerei* sp. nov., Kouvola, Finland **B** paratype female of *Scenopinusjerei* sp. nov., Kouvola, Finland **C** male of *Scenopinusfenestralis*, Liperi, Finland **D** female of *Scenopinusfenestralis*, Liperi, Finland **E** male of *Scenopinusvitripennis*, Württburg, Germany. Photograph by Susanne Leidenroth **F** female of *Scenopinusvitripennis*, Warszawa, Poland. Photograph by D. Schimrosczyk **G** female of *Scenopinusglabrifrons*, Ockstadt, Germany. Scale bar: 500 mm.

**Figure 2.**
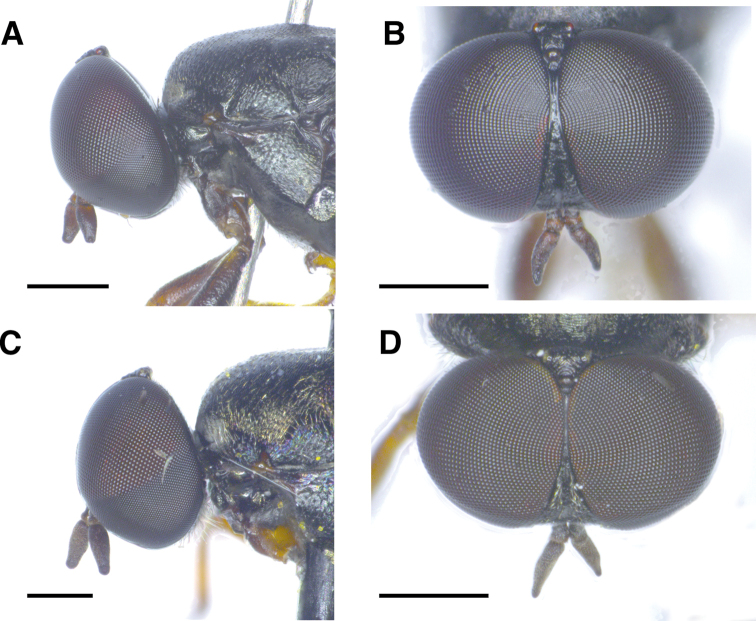
Male heads of *Scenopinus* species **A** lateral view of holotype *Scenopinusjerei* sp. nov., male head **B** frontal view of holotype *Scenopinusjerei* sp. nov., head **C** lateral view of *Scenopinusfenestralis* male head **D** frontal view of *Scenopinusfenestralis* male head. For the illustrations of male heads of *S.vitripennis* and *S.glabrifrons*, see Krivosheina (1981). Scale bar: 500 mm.

**Figure 3.**
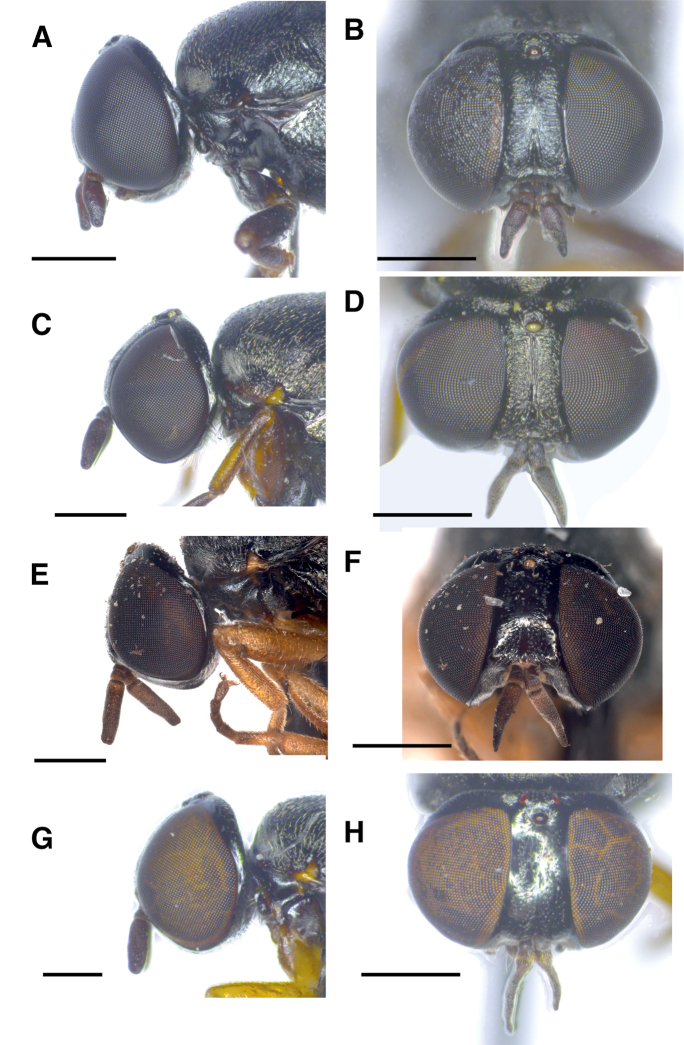
Female heads of *Scenopinus* species **A** lateral view of paratype *Scenopinusjerei* sp. nov., female head **B** frontal view of paratype *Scenopinusjerei* sp. nov., head **C** lateral view of *Scenopinusfenestralis* female head **D** frontal view of *Scenopinusfenestralis* female head **E** l ateral view of *Scenopinusvitripennis* female head. Photograph D. Schimrosczyk **F** frontal view of *Scenopinusvitripennis* female head. Photograph D. Schimrosczyk **G** lateral view of *Scenopinusglabrifrons* female head **H** frontal view of *Scenopinusglabrifrons* female head. Scale bar: 500 mm.

**Figure 4.**
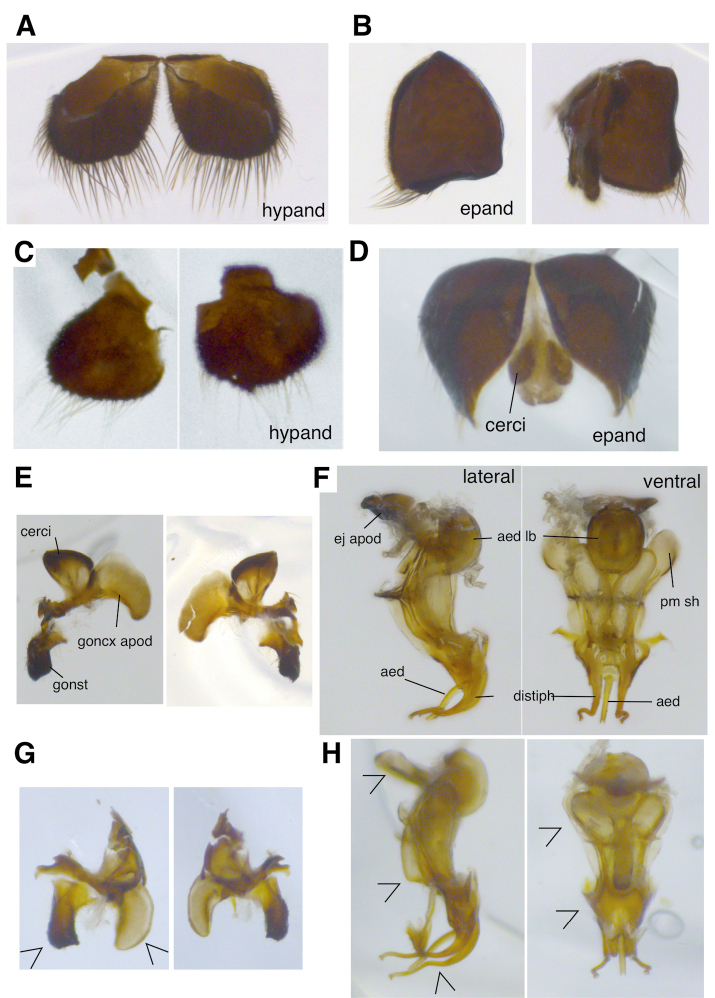
*Scenopinus* male terminalia **A** hypandrium of *Scenopinusjerei* sp. nov. paratype **B** epandrium of *Scenopinusjerei* sp. nov. paratype **C** hypandrium of *Scenopinusfenestralis***D** epandrium of *Scenopinusfenestralis***E** terminal segments of *Scenopinusjerei* sp. nov. paratype **F** aedeagus of *Scenopinusjerei* sp. nov. paratype **G** terminal segments of *Scenopinusfenestralis***H** aedeagus of *Scenopinusfenestralis*. *aed* – aedeagus; *aed lb*– aedeagal lobe; *distph* – distiphallus; *ej apod* – ejaculatory apodeme; *epand* – epandrium; *goncx apod* – gonocoxal apodeme; gonst – gonostylym; *hypand* – hypandrium; *pm sh* – parameral sheath. For the illustrations of male terminalia of *S.vitripennis* and *S.glabrifrons*, see Krivosheina (1981).

Figures 1A, B , 2 , 3A, B , 4A, B , 4E, F

#### Type material.

***Holotype*** (1♂): Finland, Sa: Kouvola, 674–679:347–350 [60.7686–61.2184, 26.4495–27.0000] / e.l. 2018 ex *Strixaluco* nest box. / M. Mutanen leg. // *Scenopinusjerei* sp. nov. Pohjoismäki & Haarto 2021 / (Diptera: Scenopinidae) / J. Pohjoismäki det. // HOLOTYPE [red label] [MZH] ***Paratypes***: 1♂ (dissected, DNA barcoded JP2020-S1), 1♀, same collection data; // *Scenopinusjerei* sp. nov. Pohjoismäki & Haarto 2021 / (Diptera: Scenopinidae) / J. Pohjoismäki det. // PARATYPE [yellow label] [MZH]; 1♂: Finland, EP: Isokyrö/ Orisberg 6983:[3]265 [62.8744, 22.3795]/ 7.7.1999/ A. Haarto leg.// SCENOPINIDAE/ *Scenopinus*/ *vitripennis* Meig./ det. A. Haarto 1999// PARATYPE/ Diptera: Scenopinidae/ *Scenopinusjerei*/ Pohjoismäki & Haarto 2021 [red label] [AHC]; 1♂: Finland, ES [Etelä-Savo]: Rantasalmi/ Korhola 68720:[3]5802 [61.9458, 28.5278] / 27.6.2006/ A. Haarto leg.// SCENOPINIDAE/ Scenopinus/ sp./ det. A. Haarto 2006// PARATYPE/ Diptera: Scenopinidae/ *Scenopinusjerei*/ Pohjoismäki & Haarto 2021 [red label] [AHC]; 2♂: Finland, Kb: Ilomantsi/ Kelovaara 70008:36826 [63.0638, 30.6144]/ 24.7.2021/ J. Pohjoismäki leg. // PARATYPE/ Diptera: Scenopinidae/ *Scenopinusjerei*/ Pohjoismäki & Haarto 2021 [yellow label] [JPC]; 1♀: Finland, ES: Rantasalmi/ Korhola 68720:5802 [61.9458, 28.5278]/ 29.6.2006/ A. Haarto leg.// SCENOPINIDAE/ Scenopinus/ sp./ det. A. Haarto 2006// PARATYPE/ Diptera: Scenopinidae/ *Scenopinusjerei*/ Pohjoismäki & Haarto 2021 [red label] [AHC]; 1♀: Finland, Kb: Liperi/ Viinijärvi 6951:3615 [62.6451, 29.2425] / e larva 2013/ Ali Karhu leg.// linnunpönttö [nest box]// SCENOPINIDAE/ *Scenopinus*/ sp./ det. A. Haarto 2014/ AHa14–000891// PARATYPE/ Diptera: Scenopinidae/ *Scenopinusjerei*/ Pohjoismäki & Haarto 2021 [red label] [AHC]; 1♀: Finland, Kb: Liperi/ Käsämä suo 6950:3619 [62.6349, 29.3197]/ 26.-28.6.2013/ Ali Karhu leg.// SCENOPINIDAE/ *Scenopinus*/ sp./ det. A. Haarto 2020/ AHa20–000473// PARATYPE/ Diptera: Scenopinidae/ *Scenopinusjerei*/ Pohjoismäki & Haarto 2021 [red label] [MZT].

#### Diagnosis.

*Scenopinusjerei* sp. nov. belongs to the *S.fenestralis* group and is easily recognisable from the other species in this group based on the contrasting colour differences between the femora and the yellow to orange tibiae. The coxae as well as the knob of the halteres are always uniformly black or dark brown, similar to the colour of the thorax.

#### Description.

**Male** (Figs 1A; 2A, B; 4A, B, F, G) (characters in square brackets refer to the holotype). Body length: [4.1]–4.9 mm (n = 6) [dried specimens, fresh specimens are longer).

***Head*** (Fig. 2A, B). Black with greasy-looking shine, including oral margin and occiput, apart from weak grey microtomentum around occipital foramen, mouth edge and antennal base; semi-circular, height 1.5–1.7 [1.6] (n = 6) × its maximum width in lateral view. Antennal insertion slightly below mid eye level. Antenna dark brown with pedicel and anterior part of flagellomere paler; scape short and subrectangular; pedicel short and cylindrical, [0.8]–1.0 (n = 6) × as long as wide, flagellomere laterally flattened, 1.8[1.9]–2.0 × as long as high, subrectangular, narrowing apically and 4.8–[5.4] (n = 6) × as long as pedicel and with subcircular, subapical, sensory pore on outer side. Eyes large and bare; fronto-orbital plates meeting at [0.25]–0.3 (n = 6) length of frons; no frontal vitta; gena reduced to narrow strip between lower eye margin and mouth edge. Diameter of ommatidia on upper half of compound eye, above antennal base, 2–3 × diameter of ommatidia on lower half. Ocellar triangle acute, distance between posterior ocelli distinctly shorter than their distance to anterior ocellus. Frons bare but patterned with minute pits. No setae or setulae on head, apart for short brown setulae at lower posterior part of gena behind mouth edge. Mouthparts, including palpus, black.

***Thorax*** (Fig. 1A). Dorsally and laterally black with greasy-looking shine. Scutum patterned with small rugae and minute, barely distinguishable setulae. Pleura with similar patterning but with more distinct, short, sparse, greyish to brownish setulae. Hirsuteness most developed on anepisternum, where longest setulae are approximately same length as width of flagellomere.

***Legs*** (Fig. 1A). Coxae black; femora brown and apically paler. Fore and mid tibiae [pale brown] to dirty orange, clearly paler than femora. Hind tibia otherwise of similar colour as femora but paler at base and apex. Hind coxa with thin black posterior setulae, longest setulae as long as width of coxa at its base. Femora with thin posterodorsal setulae, longest being 0.5 × as long as width of femora. Fore tibia preapically with [2]–3 ventral setulae. Mid tibia with two short, ventral, preapical setae and two adjacent setulae. Hind tibia with one ventral preapical setula and thin posteroventral setulae covering proximal half, longest of which are as long as width of tibia. Apart from aforementioned setae and setulae, all legs covered in minute setulae that provide rugous texture.

***Wings*** (Fig. 1A). Hyaline with greyish tinge. Tegula black, basicosta brownish black, wing veins brown. Petiole and knob of haltere uniformly black.

***Abdomen*** (Fig. 1A). Elongated, dorsally flattened except for domed terminal segments, black in colour, with greasy shine and covered by irregular, minute, robust setulae. Tergites 6, 7, and 8 with black marginal setulae, longest of which ca. as long as width of hind femora.

***Terminalia*** (Fig. 4A, B, E, F). Hypandrium divided to two similar subrectangular halves, posterior margin with long dense setulae (Fig. 4A). Epandrium similarly divided into subtriangular halves with long posteroventral marginal setulae (Fig. 4B). Cercus subtriangular (Fig. 4E); gonocoxal apodeme scapula-shaped and curving slightly inwards at its posterior part; gonostylym bluntly subrectangular, posteriorly concave and ending with a ventral apex. Distiphallus with long, narrow, curved apical processes, hooking outwards prior to apex (Fig. 4F). Aedeagus rod-shaped, slightly bent at middle and with a short-forked apex.

**Female** (Figs 1B, 3A, B). Differs from male as follows:

Body length: 4.3–5.7 mm (n = 4). ***Head*** (Fig. 3A, B). Frons broad, at its narrowest point 0.62–0.69 (n = 4) × as wide as an eye in dorsal view. Frons shiny black with minute longitudinal rugae on frontal stripe and transverse rugae on sides. Orbital plates smooth and shiny. Ocellar triangle equilateral. No obvious size difference between ommatidia of upper and lower half of compound eye. ***Thorax*** (Fig. 1B). Very weak whitish grey microtomentum at anterior parts of postpronotum and proepisternum. ***Abdomen*** (Fig. 1B). Dorsally flattened along its entire length. ***Terminalia***. Last visible tergite 9 bluntly triangular at its posterior edge and not divided into hemitergites.

#### DNA barcode divergence among *Scenopinus*.

**Figure 5.**
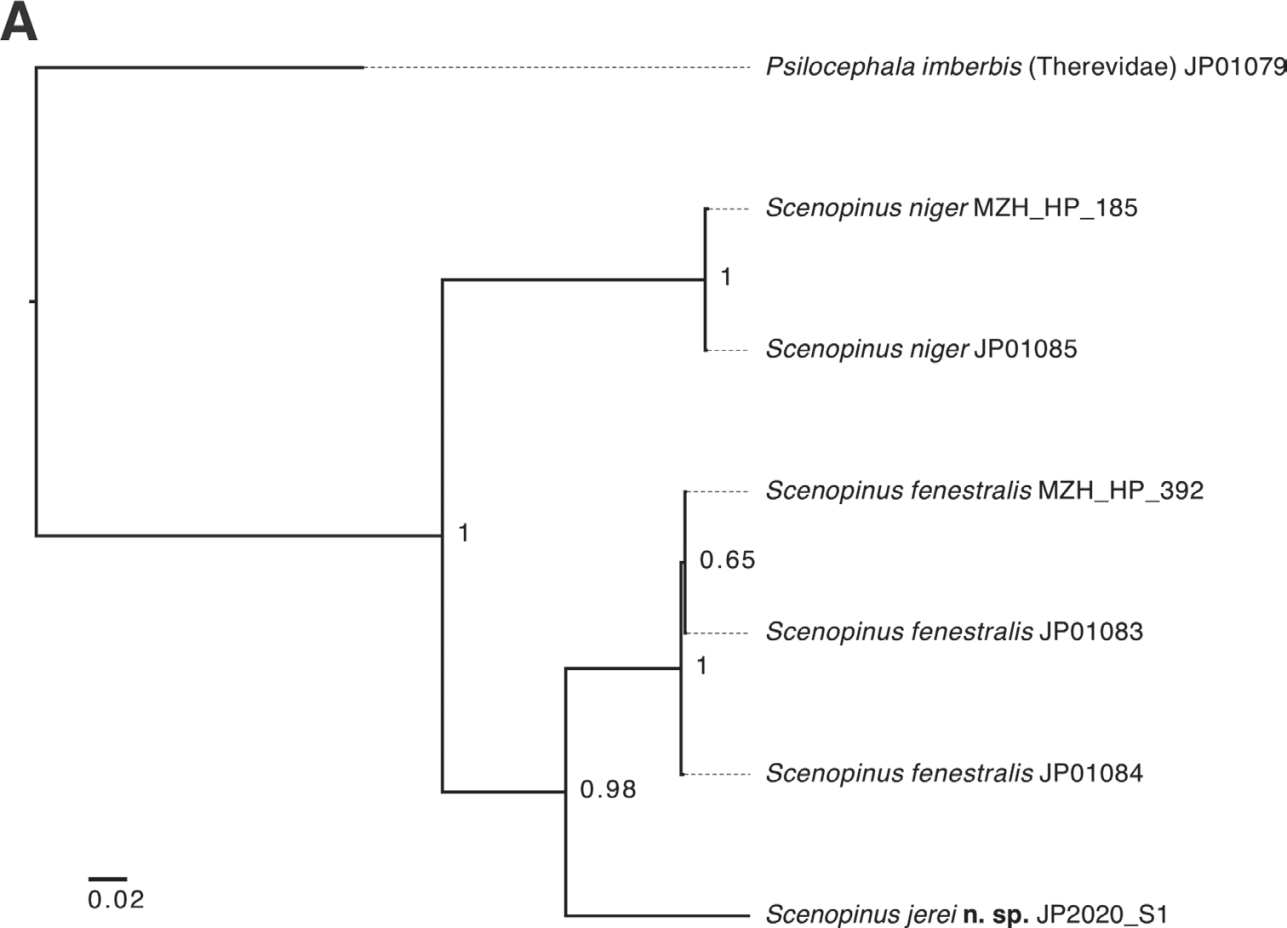
Maximum likelihood tree of *COI* sequence similarities among *Scenopinus* spp. Numbers at nodes indicate posterior probabilities and scale bar the relative sequence divergence. BOLD sample ID number is given after the species name.

*Scenopinus* spp. are poorly covered in the DNA barcode databases, such as Barcode of Life Database (BOLD, www.boldsystems.org) or GenBank. It is noteworthy that all *S.fenestralis* specimens in the databases from Europe to North America have almost identical *COI* sequences and represent the same barcode index number (BIN). The DNA barcode of *Scenopinusjerei* sp. nov. differs markedly from the other northern European species, its closest match being *Scenopinusfenestralis* from which it is separated by 12.48% sequence difference (Fig. 5). There are no other closer matches among the barcode sequences in the BOLD or GenBank.

#### Notes

on the biology and distribution of *Scenopinusjerei* sp. nov. The larvae of *Scenopinus* species are predators of other invertebrates living in dry organic substrates, such as in animal nests. In Finland, *Scenopinusjerei* sp. nov. has been collected inside sheds, attics and indoor storages as well as reared from nest boxes of birds. These rearings produced large numbers of tineid moths (Lepidoptera: Tineidea), especially *Monopislaevigella* (Denis & Schiffermüller), but also other *Monopis* spp., *Niditineastriolella* (Matsumura), and *Tinea* spp. Other insects observed from the same nest boxes included *Ceratophyllus* fleas, various beetles (Histeridae, Dermestidae) and flies (Piophilidae, Fanniidae, Heleomyzidae). Apart for two male specimens found dead on a windowsill in an attic of an old house in Kelovaara on July 24 (see type specimens), most observations are from third week of June. According to the observations of Jere Kahanpää (pers. comm.), *Scenopinusjerei* sp. nov. hibernates as full-grown larvae and the adults emerge in a couple of weeks in room temperature rearing conditions. Based on the collection locations, it is likely that *Scenopinusjerei* sp. nov. is a boreal forest specialist.

Like other *Scenopinus* spp., *Scenopinusjerei* sp. nov. is not very active flier, does not visit flowers and therefore is rarely collected by active netting or traps. Judging from the few Finnish observations, the species appears widespread in the southern and central parts of the country. We are certain that *Scenopinusjerei* sp. nov. can also be found in boreal forest biotopes in the other Nordic countries and Russia but has been until now overlooked.

#### Etymology.

This species is named after Mr. Jere Kahanpää, Helsinki, who was to first to discover that the taxon is new to science and kindly agreed with the current arrangement for its formal description.

##### Provisional key to the identification of European *Scenopinidae* species

Because the existing literature on the European species of Scenopinidae are outdated or difficult to obtain, we felt necessary to provide a key for the known European species of Scenopinidae. We must emphasise that we have been only able to examine the species with specimens listed in this paper, for which the identification key should work well. For the remainder, our approach was to go through the written species descriptions and pick features which we judged, by our collective species identification experience, to be useful for determination. To us this approach was better justified than reproducing the keys given in earlier literature, which are often difficult to follow or focus on limited number of poorly defined features. The diagnostic features for the key have been obtained from the descriptions in Kröber (1925), Trojan (1956), Kelsey (1969), Krivosheina (1981), Narchuk (1988), and Carles-Tolrá (2001). Fortunately, the European species separate into three easily recognisable species groups, each with relatively few species. The species groups appear in the key in alphabetical order, enabling fast navigation when one is familiar with the groups. Although result appears satisfactory, the key might not capture all the variations seen within each species and we strongly encourage DNA barcoding of specimens for future reference. In any case, we hope that this key can form a basis for forthcoming work with this interesting family of flies.

### Key to genera

**Table:** 

1	Cell r_5_ closed and petiolate (Fig. 6B).	***Caenoneuranigra* Kelsey, 1969 (Spain)**
–	Cell r_5_ open (Fig. 6C). Genus *Scenopinus*	**2**

### Genus *Scenopinus*

**Table:** 

2	Flagellomere short and stout, ~ 1.5 × as long as wide, pear-shaped by distinctly narrowing towards the tip	***brevicornis* group 9**
–	Flagellomere elongated, if apically narrowing then > 1.7 × as long as wide	**3**
3	Generally small in size (< 3 mm); R_4_ branching from R_5_ beyond the middle of cell r_5_ (Fig. 6D); aedeagus in males not concealed by the epandrium	***albicinctus* group 4**
–	Larger and more robust flies; R_4_ branching from R_5_ at or before the middle of cell r_5_ (Fig. 6C); aedeagus in males concealed by the epandrium	***fenestralis* group 13**

### *albicinctus* group

**Table:** 

4	Thorax and abdomen shining black; wings hyaline; legs black with orange-brown tarsi.	***Scenopinusphaidimos* Kelsey, 1969** (Turkey)
–	Other combination of features	**5**
5	Thorax and legs with moderately long golden setulae; thorax black-brown; humeral callus red-brown with yellow posterior patch; abdomen in males with narrow white bands on the posterior margin of tergites 3 and 4	***Scenopinuscanarius* Kelsey, 1969** (Canary Islands)
–	No long golden setulae on thorax and legs.	**6**
6	Tarsal claws as long as the last tarsal segment; single white band on the abdomen; antenna black; halter knob pale.	***Scenopinusbouvieri* (Seguy, 1921)** (central and southern Europe)
–	Tarsal claws shorter than the last tarsal segment.	**7**
7	Frons white; thorax dark with wide yellow band [spot?] extending from the humeral area to the posterior margin of the scutellum; male abdomen with 3 distinct white bands, female abdomen black with yellow spot on tergite 2	***Scenopinusalbicinctus* (Rossi, 1794)** (southern Europe)
–	Other combinations of features.	**8**
8	Thorax black with rugous texture and patch of microtomentum above the humeral callus; humeral callus red-brown with yellow posterior patch; wings opaque brown; halters and legs uniformly brown; abdomen in males with narrow white bands on the posterior margin of tergites 3 and 4, in females uniform dark red-brown	***Scenopinusbulbapennis* Kelsey, 1969** (Spain)
–	Uniformly matt grey-brown species; thorax with two dark longitudinal stripes; tergite margins of the abdomen with diffuse brown-yellow banding; legs dark brown with paler knees and tarsi	***Scenopinusoldenbergi* (Kröber, 1913)** (African species imported to Europe?)

### *brevicornis* group

**Table:** 

9	Abdomen with broad white bands on the posterior margins of tergites 2–6, giving it a solid white appearance.	***Scenopinusgobiensis* Kelsey, 1981** (Hungary, eastern Palearctic)
–	Not as above	**10**
10	Wing opaque white; a very small species (1.5 mm)	***Scenopinushalteralis* Frey, 1936** [only males known] (Canary Islands)
–	Wing opaque brown; larger species	**11**
11	Supra-alar callus (anteriorly to wing base) orange with a distinct dark brown, warty protuberance in both sexes	***Scenopinusverrucosus* Carles-Tolrá, 2001** (Spain)
–	No warty protuberance on supra-alar callus.	**12**
12	Humeral callus red-brown with white posterior border; abdomen in males black-brown with white band on the posterior margin of tergites 3–5, in females uniform red-brown	***Scenopinusunifasciatus* (Kröber, 1913)** (eastern Mediterranean)
–	Humeral callus uniform black-brown; male abdomen brown.	***Scenopinusretuertensis* Carles-Tolrá, 2001** (Spain)

### *fenestralis* group

**Table:** 

13	Thorax and abdomen red brown with short white setulae; posterior margin of humeral callus orange; legs brown except for yellow tarsi	***Scenopinusefflatouni* Kelsey, 1969** [only females known] (Andorra)	
–	Not as above	**14**	
14	Cell cu narrow (Fig. 6E); wings opaque brown; male with white-banded abdomen and distinct grey microtomentum on mesonotum	***Scenopinusgriseus* (Kröber, 1913)** (south-eastern Europe)	
–	Cell cu wide (Fig. 6C).	**15**	
15	Legs bicoloured; coxae and femora black or dark brown, apically paler; fore and mid tibiae orange to pale brown; haltere entirely black	***Scenopinusjerei* sp. nov.** (Finland [northern Europe])	
–	No colour difference between femora and tibia; haltere variable, from white to black.	**16**	
16	Legs unicolourous black or dark brown (in doubtful cases, as dark as ground	colour of thorax), only tarsi paler than tibiae.	**17**
–	Legs orange to pale brown (in doubtful cases, paler than ground colour of thorax)	**18**	
17	Wings black. Head subrectangular in lateral profile, antennae ~ 1/2 as long as the frons. In males, the apex of the hind tibia wider than the femora. Eyes not touching in either sex	***Scenopinusniger* (De Geer, 1776)** (northern and central Europe)	
–	Wings clear with yellow veins. Head hemispherical in profile. Eyes touching in males, widely separated in females	***Scenopinuslesinensis* Strobl, 1902** (southern Europe)	
18	Lower part of head with thin white microtomentum, genal setulae mostly dark.	Flagellomere subrectangular and apically narrowing. Female frons with distinct rugae	***Scenopinusfenestralis* (Linnaeus, 1758)** (cosmopolitan)
–	Lower part of head around the mouth edge to the antennal insertion with dense white microtomentum, genal setulae pale. Flagellomere cylindrical, not (or scarcely) narrowing apically. Female frons smooth and shiny.	**19**	
19	The knob of haltere brown, as dark as its stem	***Scenopinusvitripennis* Meigen, 1824** (central and eastern Europe)	
–	The knob of haltere white, paler than its stem	***Scenopinusglabrifrons* Meigen, 1824** (central and southern Europe, cosmopolitan)	

## Discussion

### Survey of candidate species among the *Scenopinusfenestralis* group

*Scenopinusjerei* sp. nov. was originally confused with *Scenopinusvitripennis* (Haarto 2000; Kahanpää and Winqvist 2005). The mistake occurred because *Scenopinusjerei* sp. nov. easily keys out as *S.vitripennis* using the key in Kelsey (1969), due to the dark halteres and relatively smooth female frons. As pointed out by Kelsey (1969), *S.vitripennis* was treated as a synonym of *S.glabrifrons* by many authors prior to Trojan (1956), who established it as a valid species based on female characters. In retrospect this conclusion is problematic, as the original type specimen of Meigen was a male and is presumed lost. At the time of Kelsey’s work (1969), the type was the only reported male specimen of the species. Interestingly, there is one male identified in 1964 by Kelsey as *S.vitripennis* in SMNS. It is unknown to us why Kelsey did not include this specimen in his work on world Scenopinidae. The specimen is only 3.4 mm long, dark, has entirely grey-brown legs and, apart for some dirt and broken antennae, is in good condition (Fig. 1E). The colour of the legs, including the pale coxae, and general appearance of the specimen does not agree with our concept of *Scenopinusjerei* sp. nov. and neither does the examined female specimen in the ZIM collection (Fig. 1F). Notably, the halteres of these specimens are grey or reddish brown, much paler than the thorax, whereas those of *Scenopinusjerei* sp. nov. are black or as dark as the thorax, similar to the situation in *S.niger*.

The original description of *Scenopinusvitripennis* by Meigen (1824:115) is brief but provides sufficient information: “*Schwartz; Beine gelbroth* [sic]; *Schwinger braun; Kopf unten weiß; Flügel glasshelle* [sic]. *Niger; pedibus rufis; halteribus fuscis; clava subtus alba; alis hyalinis.*” [Black; legs yellow-red; halteres brown, underside of the head white; wings hyaline.]

Meigen’s description of the colouration of the legs as well as the underside of the head make it clear that *S.vitripennis* is not conspecific with our *Scenopinusjerei* sp. nov. However, these features are also not evident in the small, dark male specimen in the SMNS collection either. It may be that Kelsey disregarded this specimen from his work for the same reason. In fact, the male of *S.vitripennis* was later redescribed and illustrated by Krivosheina (1981). The shape of the antennae, terminal sternites as well as the visible genitalia features in Krivosheina’s illustrations differ markedly from those of *Scenopinusjerei* sp. nov. However, the comparison with *S.glabrifrons* is not very detailed in Krivosheina’s work. We also note that the female specimen in MIZ, collected and identified by Trojan, is very similar to *S.glabrifrons* in general appearance, including the flagellomere, which is cylindrical and parallel sided, compared to the basally more robust, apically narrowing flagellomere *Scenopinusjerei* sp. nov.

For some reason, the male genitalia of Scenopinidae have been traditionally dissected only partially, the only visible parts being the proximal parts of the aedeagus as well as the terminal segments, which makes the comparison of the published illustrations prone to interpretation errors. When fully dissected, the aedeagus has very distinct, species-specific features (Fig. 4), which could be better utilised for species identification. Unfortunately, *S.vitripennis* is very rare in collections, and clearly more work is needed to fully understand the extent of interspecific variation between it and *S.glabrifrons*. These future efforts should also focus on applying modern sequencing methods that allow the genotyping of old museum specimens (Staats et al. 2013; Prosser et al. 2016; Nakahama 2021).

To further validate our interpretation of the new species status of *Scenopinusjerei* sp. nov., we also checked the potential candidates among the known species of *Scenopinus* outside Europe. Since the revision of the world Scenopinidae by Kelsey (1969), relatively few *Scenopinus* species have been described (Pape and Thompson 2021), most of which can be excluded by their exotic location. However, as several Eastern Palearctic species in other families extend their range to Finland (e.g., Cannings and Kahanpää 2013; Stuke et al. 2020; Pohjoismäki and Bergström 2021), we wanted to rule out the following species from the Russian Far East:

- Scenopinus mariensis Kelsey, 1981. This species is close to S. lesinensis but has notable colour patterning on the thorax.
- Scenopinus sibiricus Krivosheina, 1982. Wings dark brown, tibiae black. Eyes separated by a frontal stripe in males. The species is morphologically close to the Nearctic Scenopinus aquelonius Kelsey, 1969, S. breviterminus Kelsey, 1969, and S. undulafrons Kelsey, 1969.
- Scenopinus ussuriensis Krivosheina, 1981. Illustrations of the antenna shape and male genitalia are dissimilar to those of Scenopinus jerei sp. nov.
- Scenopinus zhelochovtsevi Krivosheina, 1982. Legs uniform in colour. Illustrations of the antenna shape and male genitalia are dissimilar to those of Scenopinus jerei sp. nov.

No potential candidates were found among the known Nearctic species.

### Survey of the old *Scenopinusfenestralis* synonyms

*Scenopinusfenestralis* is a common, variable, and widespread species. It also lacks clear morphological features, such as strong setae that are used as diagnostic characters for many fly groups, making it difficult to devise generalised descriptions or identification keys for both sexes. For example, the colour of the halteres can vary from dark brown to white and the colour of the legs from pale brown to orange. It is probable that developmental factors and possibly age of the fly play a big role in the morphological variation. Among others, it is known that hoverflies (Diptera: Syrphidae) developing in cool and humid conditions are typically darker than the ones developed in warm and dry conditions (e.g., Ottenheim et al. 1996). Similarly, small specimens developed under insufficient nutrition tend to be darker and morphologically more plain than larger specimens, which could also explain the aberrative habitus of the putative *S.vitripennis* male specimen in SMNS, mentioned earlier.

**Figure 6.**
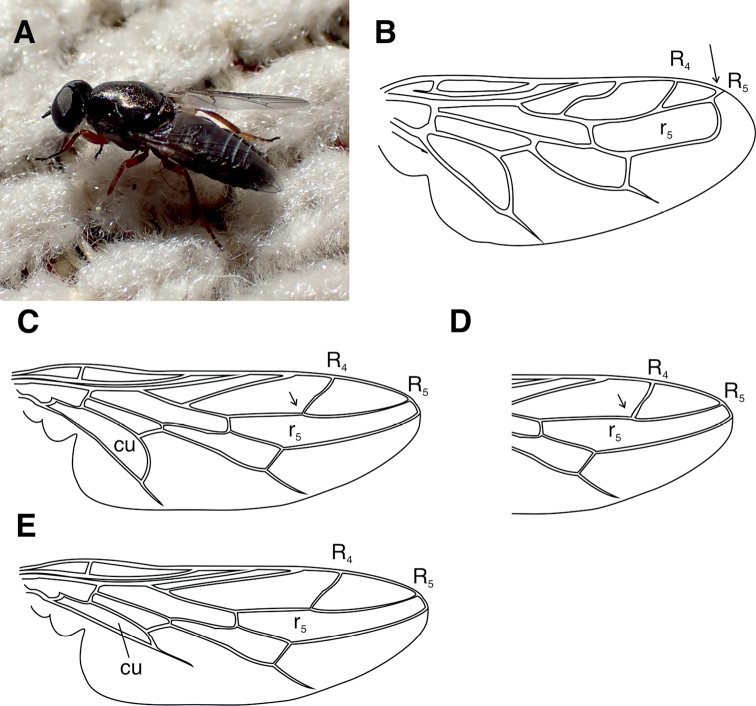
Identification of Scenopinidae**A** male *Scenopinusfenestralis*. Hanko, Finland, June 22, 2021. Note the three stripes on the abdomen caused by the white integument protruding between the tergites. These should not be confused with white bands of microtomentum on tergites of some *Scenopinus* species. Photograph by J. Pohjoismäki **B** illustration of *Caenoneura* wing. Arrow pointing the petiole on r_5_. Drawn after Kelsey (1969)**C** generic *Scenopinus* wing. Modified from Winterton and Gaimari (2017)**D** wing venation in *albicinctus*-group with R_4_ branching from R_5_ beyond the middle of cell r_5_ (arrow; compare with C) **E** Wing of *Scenopinusgriseus* (Kröber) with narrow cu cell. Drawn after Narchuk (1988).

This intraspecies variation has contributed to the wealth of synonyms for *S.fenestralis*, which raised the question if some of the names could correspond to our concept of *Scenopinusjerei* sp. nov. However, checking all the old, synonymised type specimens dispersed over several collections would have been impossible. Fortunately, the colouration of *Scenopinusjerei* sp. nov. legs and halteres are so distinct that we were able to validate these diagnostic characters even from the quite brief old descriptions. Pape and Thompson (2021) list the following synonyms for *S.fenestralis*, whose original descriptions differ from the characters of *Scenopinusjerei* sp. nov. as indicated:

- Musca tarda Linnaeus, 1761 – White halteres, yellow legs.
- Musca saltitans Scopoli, 1763 – White halteres, red legs.
- Musca spoliata Scopoli, 1763 – Male specimen of the latter.
- Musca senilis Fabricius, 1794 – Legs yellow-red, head white from below.
- Atricha fasciatus Schrank, 1803 – Greyish specimen with milk-white bands on the abdomen. While the abdominal banding sounds like an unusual feature for S. fenestralis, we have observed that the soft white integument can be protruding between the tergites in newly hatched Scenopinus specimens, giving the abdomen a banded appearance (Fig. 6A). Legs entirely olive brown.
- Scenopinus pallipes Say, 1823 – White halteres, yellow legs.
- Scenopinus domesticus Meigen, 1824 – Legs yellow-red, head white from below.
- Scenopinus sulcicollis Meigen, 1824 – Legs yellow-red, head white from below, halteres white.
- Scenopinus scutellatus Macquart, 1843 – Halteres white, scutellum yellow.
- Scenopinus furcinervis Zetterstedt, 1844 – Legs fully yellow.
- Scenopinus fuscinervis Schiner, 1860 – The name is not mentioned in Schiner (1860).
- However, Kelsey attributes the synonymy to Schiner (1862), where S. fuscinervis Zetterstedt is given as a synonym of S. fenestralis. It is obvious that in this context, S. fuscinervis is a misspelling of S. furcinervis. In fact, the spelling is later corrected in Schiner (1864).
- Scenopinus graminicola Zetterstedt, 1859 – Halteres white.
- Scenopinus nigroscutellatus Frey, 1945 – From Azores, halteres white.

Based on this survey, we are confident that the species presented here as *Scenopinusjerei* sp. nov. is not among the accepted species nor hidden among the synonyms of *S.fenestralis*. We hope that the species discovery reported here, together with the provisional indentification key we have provided, will encourage more research towards this exciting but poorly known family of flies.

## Supplementary Material

XML Treatment for Scenopinus jerei
